# Vaccinomic approach for novel multi epitopes vaccine against severe acute respiratory syndrome coronavirus-2 (SARS-CoV-2)

**DOI:** 10.1186/s12865-021-00412-0

**Published:** 2021-03-25

**Authors:** Yassir A. Almofti, Khoubieb Ali Abd-elrahman, Elsideeq E. M. Eltilib

**Affiliations:** 1 Department of Molecular Biology and Bioinformatics, College of Veterinary Medicine, University of Bahri, Khartoum, Sudan; 2 Department of Pharmaceutical Technology, College of Pharmacy, University of Medical Science and Technology (MUST), Khartoum, Sudan

**Keywords:** SARS CoV-2, Spike S protein, orf1ab polyprotein, Multiepitopes vaccine, B-lymphocytes, T-lymphocytes

## Abstract

**Background:**

The spread of a novel coronavirus termed severe acute respiratory syndrome coronavirus-2 (SARS-CoV-2) in China and other countries is of great concern worldwide with no effective vaccine. This study aimed to design a novel vaccine construct against SARS-CoV-2 from the spike S protein and orf1ab polyprotein using immunoinformatics tools. The vaccine was designed from conserved epitopes interacted against B and T lymphocytes by the combination of highly immunogenic epitopes with suitable adjuvant and linkers.

**Results:**

The proposed vaccine composed of 526 amino acids and was shown to be antigenic in Vaxigen server (0.6194) and nonallergenic in Allertop server. The physiochemical properties of the vaccine showed isoelectric point of 10.19. The instability index (II) was 31.25 classifying the vaccine as stable. Aliphatic index was 84.39 and the grand average of hydropathicity (GRAVY) was − 0.049 classifying the vaccine as hydrophilic. Vaccine tertiary structure was predicted, refined and validated to assess the stability of the vaccine via Ramachandran plot and ProSA-web servers. Moreover, solubility of the vaccine construct was greater than the average solubility provided by protein sol and SOLpro servers indicating the solubility of the vaccine construct. Disulfide engineering was performed to reduce the high mobile regions in the vaccine to enhance stability. Docking of the vaccine construct with TLR4 demonstrated efficient binding energy with attractive binding energy of − 338.68 kcal/mol and − 346.89 kcal/mol for TLR4 chain A and chain B respectively. Immune simulation significantly provided high levels of immunoglobulins, T-helper cells, T-cytotoxic cells and INF-γ. Upon cloning, the vaccine protein was reverse transcribed into DNA sequence and cloned into pET28a(+) vector to ensure translational potency and microbial expression.

**Conclusion:**

A unique vaccine construct from spike S protein and orf1ab polyprotein against B and T lymphocytes was generated with potential protection against the pandemic. The present study might assist in developing a suitable therapeutics protocol to combat SARSCoV-2 infection.

## Background

A novel coronavirus termed severe acute respiratory syndrome related coronavirus-2 or SARS-CoV-2 was identified in China in late 2019. The virus is the causative agent of coronavirus disease 2019 (COVID-19) and is contagious through human-to-human transmission [1, 2]. The disease is characterized by severe respiratory illness with symptoms of fever, cough, and shortness of breath and significant mortality, particularly among patients over the 60 years of age and in those suffering from chronic conditions such as diabetes and hypertension [3, 4]. SARS-CoV-2 was first reported in Wuhan, Hubei Province, in China, and swiftly spread all over China and other countries [4]. The causative agent of the outbreak was identified as Betacoronavirus with a genomic sequence closely related to that of the severe acute respiratory syndrome (SARS) coronavirus from 2003, hence the name SARS-CoV-2 [5–8]. The disease had become pandemic and globally spread to many countries and territories, including community transmission in countries like the United States, Germany, France, Spain, Japan, Singapore, South Korea, Iran and Italy with high significant morbidity and mortality rates [9].

SARS-CoV-2 is a positive-strand RNA virus that belongs to the group of Betacoronaviruses. The genome of the virus consists of 29,700 nucleotides with 79.5% sequence similarity to SARS-CoV. The virus encodes multiple structural and non-structural proteins [4, 10]. The orf1ab polyprotein is nonstructural protein at the 5 prime end of the viral genome constitutes two third of the viral proteome and encodes for 15 or 16 non-structural proteins. The 3 prime end of the genome encodes four major structural proteins, including the spike (S) protein, nucleocapsid (N) protein, membrane (M) protein, and the envelope (E) protein in addition to nonstructural proteins including orf3a, orf8, orf7a, orf7b, orf6 and orf10 [10, 11].

Like SARS-CoV, SARS-CoV-2 binds to the receptor angiotensin converting enzyme 2 (ACE2) on the host cell via the receptor binding domain (RBD) on the spike S protein of the virus [7, 11]. The spike S protein of SARS-CoV-2 is type I transmembrane glycoprotein with predicted length of 1273 amino acids. Moreover it comprises the major antigenic determinants that induce neutralizing antibodies [12, 13]. SARS-CoV and SARS-CoV-2 demonstrated 89.8% sequence identity in the S2 subunits of their spike (S) protein, which mediate the membrane fusion process. Moreover the S1 subunits of both viruses utilized human angiotensin-converting enzyme 2 (hACE2) as the receptor to infect human cells [7, 14]. Specific amino acids sequence region within the spike S proteins, termed receptor binding domain (RBD), is considered as a functional domain responsible for virus binding to the target cell receptor [15–17]. Most importantly, the RBD present in S1 subunit of spike S protein of SARS-CoV-2 has 10 to 20 fold high affinity to bind to the target cell receptor than that of SARS-CoV. This high affinity may contribute to the higher infectivity and transmissibility of SARS-CoV-2 compared to SARS-CoV [18, 19]. In addition to that the most existing vaccine candidates against SARS CoV were based on the spike S protein and RBD region [12, 13, 15, 20, 21].

The nonstructural orf1ab gene is the largest gene segment of SARS-CoV-2 and it constitutes orf1a and orf1b [2]. The replicase orf1ab is cleaved by papain-like protease (PLpro) and 3C-like protease (3CLpro). Orf1ab is cleaved into many nonstructural proteins (NSP1-NSP16) [2, 22]. Moreover it was shown that proteins or protein domains encoded in orf1ab may serve specific roles in virulence, virus–cell interactions and/or alterations of virus–host response [23]. This indicated that orf1ab polyprotein plays an important role in the virus pathogenesis distinct from or in addition to functions directly involved in viral replication. Recent reverse genetic study confirmed that proteins of orf1ab polyprotein may be involved in cellular signaling and modification of cellular gene expression, as well as virulence. Moreover it has become clear that NSP order, expression level, and proteolytic processing may constitute distinct virulence alleles [23]. Furthermore it was suggested that the orf1ab polyproteins, notably NSP3, may interact with multiple structural and nonstructural proteins, as well as with regulatory sequences in viral RNA [23].

To control SARS-CoV-2 infection, several old drugs such as chloroquine phosphate provided slight positive effect on the treatment of the novel coronavirus pneumonia [24, 25]. Vaccination process is significantly increased to develop a vaccine against pandemic SARS-CoV-2, including the development of several RNA and DNA vaccines, recombinant protein vaccines and cell-culture-based vaccines [9]. The mRNA vaccines are a new type of vaccines to protect against infectious diseases. Recently Food and Drug Administration (FDA) has authorized the emergency use of the Pfizer-BioNTech COVID-19 Vaccine (BNT162b2) to prevent COVID-19 in individuals 16 years of age and older under an emergency use authorization given in two doses 3 weeks apart. However this vaccine showed allergic reactions such as difficulty in breathing, welling of face and throat, fast heartbeat, skin rashes, dizziness and weakness [26, 27]. Another vaccine by ModernaTX, Inc. (mRNA-1273) is recommended for people aged 18 years and older. But the vaccine also showed side effects that usually started within a day or two of getting the vaccine [26, 27].

The advances made in the field of immunoinformatics tools coincided with the knowledge on the host immune response leads to new disciplines in vaccine design against diseases via computer in silico epitope predictions. The epitopes driven vaccine is a new concept that is being successfully applied in multiple studies, particularly to the development of vaccines targeting conserved epitopes in variable or rapidly mutating pathogens [28–30]. Therefore, as the genome and proteome sequences of SARS-CoV-2 is swiftly made available [6–8], this study aimed to use immunoinformatics approach to design multi epitopes vaccine against SARS-CoV-2 infection from the structural spike S protein and the nonstructural orf1ab polyprotein.

## Results

### Sequences alignment

**Fig. 1:**
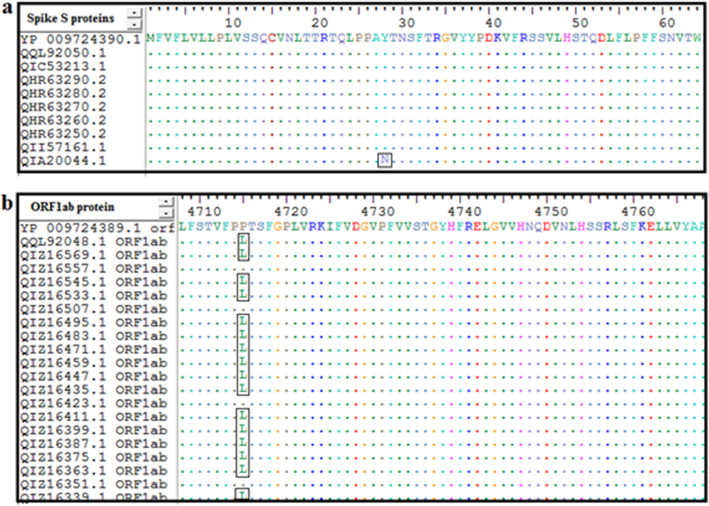
Multiple sequence alignment (MSA) of the retrieved strains of **a** spike S protein and **b** orf1ab polyprotein using Bioedit software and ClustalW. Letters within the rectangular indicated the non-conserved areas and dots indicated the conserve regions

Sequence alignment of all retrieved strains was performed using ClustalW that presented by Bioedit software. As shown in Fig. 1, the retrieved sequences of the spike S protein and orf1ab polyprotein including those of the new variant strain of Britain (SARS-CoV-2 VUI 202012/01 (MW450666.1) demonstrated high level of epitopes conservancy. The new variant strain was included since it is important to design a vaccine combating the infections from wild-type and mutant forms of SARS-CoV2. The conserved regions from both proteins were recognized by identity of amino acid sequences among the retrieved sequences. All the predicted epitopes that showed 100% conservancy in the tools of B and T lymphocytes were included for further analysis while the non-conserved epitopes were excluded.

### B-cell epitopes prediction

The reference sequences of the spike S protein (YP_009724390.1) and orf1ab polyprotein (YP_009724389.1) were subjected to BepiPred linear epitopes prediction, Emini Surface Accessibility prediction, Kolaskar and Tongaonkar Antigenicity prediction, Karplus and Schulz flexibility and Parker hydrophilicity prediction tools in the IEDB server. The thresholds for each prediction method for each protein were shown in Table 1. The spike S protein and orf1ab polyprotein demonstrated 33 and 178 linear conserved epitopes with different lengths, respectively. When these epitopes further analyzed by the other B cell prediction tools, only one epitope from the spike S protein and four epitopes from orf1ab were passed the B cell tools and were shown to be antigenic, non-allergic and non-toxic. These epitopes, their length and position in each protein were shown in Table 1.

**Table 1:** Predicted B cell epitopes, their antigenicity, allergenicity and toxicity from spike S protein and orf1ab polyprotein

Protein	Epitope^a^	Start	End	Length	Emini surface accessibility Prediction^b^	Kolaskar & Tongaonkar antigenicity Prediction^c^	Karplus & Schulz flexibility Prediction^d^	Parker hydrophiliciy Prediction^e^	Vaxijen antigenicity^f^	Allergenicity	Toxicity
**Spike S protein**	VYDPLQPE	1137	1144	8	1.923	1.082	Flexible	1.65	0.5148	Nonallergen	Nontoxin
**orf1ab**	SLDTYPSL	2297	2304	8	1173	1066	Flexible	1.25	0.6746	Nonallergen	Nontoxin
	KSVYYTSNP	1533	1541	9	308	1045	Flexible	2.833	0.6523	Nonallergen	Nontoxin
	DASGKPVPY	2924	2932	9	1632	1046	Flexible	3.178	0.6980	Nonallergen	Nontoxin
	VKGLQPSVGPKQ	6600	6611	12	1136	1066	Flexible	2.408	1.0641	Nonallergen	Nontoxin

^a^Bepipred linear epitope prediction threshold for both proteins was 0.350

^b^Emini surface accessibility prediction threshold for both proteins was 1.000

^c^Kolaskar & tongaonkar antigenicity prediction threshold for spike S protein and orf1ab was 1.041 and 1.044, respectively

^d^Karplus & schulz flexibility prediction threshold for spike S protein and orf1ab was 0.993 and 0.988, respectively

^e^Parker hydrophiliciy prediction threshold for spike S protein and orf1ab was 1.238 and 1.127, respectively

^f^Vaxijen antigenicity threshold for both proteins was 0.4

### Cytotoxic T lymphocytes epitopes prediction

The reference sequences of the spike S protein (YP_009724390.1) and orf1ab polyprotein (YP_009724389.1) were analyzed using IEDB MHC-1 binding prediction tools to predict T cell epitopes interacting with MHC Class I alleles. This was performed based on Artificial Neural Network (ANN) with half-maximal inhibitory concentration (IC50) ≤ 100. A total of 218 and 358 epitopes were predicted interacting with different MHC-1 alleles from the spike S protein and orf1ab polyprotein, respectively. The antigenic, nonallergic, nontoxic epitopes that provided high population coverage and high allelic interactions with MHC-1 alleles were elected as vaccine candidates. Accordingly five epitopes from the spike S protein and seven epitopes from the orf1ab were chosen as vaccine candidates. These epitopes, their position and population coverage were provided in Table 2.

**Table 2:** The predicted T cytotoxic cells epitopes, their antigenicity, allergenicity, toxicity and the population coverage from spike S protein and orf1ab polyprotein

Protein	Epitopes	Start	End	Vaxijen antigenicity (0.4)	Allergenicity	Toxicity	Population coverage
**Spike S protein**	FTISVTTEI	718	726	0.8535	Nonallergen	Nontoxin	52.54%
	FVFLVLLPL	2	10	0.8601	Nonallergen	Nontoxin	48.45%
	VVFLHVTYV	1060	1068	1.5122	Nonallergen	Nontoxin	48.45%
	VRFPNITNL	327	335	1.1141	Nonallergen	Nontoxin	41.68%
	^a^FAMQMAYRF	898	906	1.0278	Nonallergen	Nontoxin	39.96%
**orf1ab**	^a^VMYASAVVL	3683	3691	0.4778	Nonallergen	Nontoxin	57.06%
	SLIYSTAAL	2242	2250	0.452	Nonallergen	Nontoxin	51.80%
	MMISAGFSL	6425	6433	1.0248	Nonallergen	Nontoxin	51.47%
	FVMMSAPPA	1804	1812	0.4871	Nonallergen	Nontoxin	48.97%
	FLLNKEMYL	3183	3191	0.44	Nonallergen	Nontoxin	45.42%
	^a^FLLPSLATV	3639	3647	0.5954	Nonallergen	Nontoxin	40.60%
	^a^SLENVAFNV	6453	6461	1.0488	Nonallergen	Nontoxin	40.60%

^a^the overlapped epitopes between MHCI and MHCII molecules were used once in the vaccine construct as MHCI or MHCII epitopes

### Helper T lymphocytes epitopes prediction

The reference sequences of the spike S protein (YP_009724390.1) and orf1ab polyprotein (YP_009724389.1) were analyzed using IEDB MHC-II binding prediction tools to predict T cell epitopes interacting with MHC Class II alleles (HLA-DR, HLA-DQ and HLA-DP). Vast amount of epitopes were predicted interacting with different MHC II alleles from the spike S protein and orf1ab polyprotein. Multiple antigenic, nonallergic and nontoxic epitopes were predicted overlapping between MHC I and MHC II. However, only the MHC II non-overlapping epitopes were considered in this stage. Among them eight epitopes from the spike S protein and ten epitopes from the orf1ab were chosen as vaccine candidates against MHC II based on their high population coverage and high allelic interaction. These epitopes, their position and population coverage were demonstrated in Table 3.

**Table 3:** The predicted T helper cells epitopes, their antigenicity, allergenicity, toxicity and the population coverage from spike S protein and orf1ab polyprotein

Protein	Core peptide (Epitope)	Peptide	Start	End	Vaxijen antigenicity (0.4)	Allergenicity	Toxicity	Population coverage
**Spike S protein**	FELLHAPAT	FELLHAPATVCGPKK	515	529	0.5409	Nonallergen	Nontoxin	98.03%
	^a^FAMQMAYRF	FAMQMAYRFNGIGVT	898	912	1.0278	Nonallergen	Nontoxin	98.93%
	FNFSQILPD	FNFSQILPDPSKPSK	800	814	0.5831	Nonallergen	Nontoxin	99.73%
	FGAISSVLN	FGAISSVLNDILSRL	970	984	0.5435	Nonallergen	Nontoxin	98.66%
	FNATRFASV	FNATRFASVYAWNRK	342	356	0.5609	Nonallergen	Nontoxin	99.23%
	LLFNKVTLA	LLFNKVTLADAGFIK	821	835	0.615	Nonallergen	Nontoxin	99.48%
	LLQYGSFCT	LLQYGSFCTQLNRAL	753	767	0.8188	Nonallergen	Nontoxin	99.21%
	NRALTGIAV	NRALTGIAVEQDKNT	764	778	0.5302	Nonallergen	Nontoxin	99.03%
	WTFGAGAAL	WTFGAGAALQIPFAM	886	900	0.4918	Nonallergen	Nontoxin	99.29%
**orf1ab**	^a^VMYASAVVL	VMYASAVVLLILMTA	3683	3697	0.4778	Nonallergen	Nontoxin	98.28%
	^a^FLLPSLATV	FLLPSLATVAYFNMV	3639	3653	0.5954	Nonallergen	Nontoxin	94.69%
	^a^SLENVAFNV	SLENVAFNVVNKGHF	6453	6467	1.0488	Nonallergen	Nontoxin	97.84%
	FFYVLGLAA	FFYVLGLAAIMQLFF	2337	2351	0.8102	Nonallergen	Nontoxin	99.72%
	YELQTPFEI	YELQTPFEIKLAKKF	249	263	0.5468	Nonallergen	Nontoxin	98.88%
	LIYSTAALG	LIYSTAALGVLMSNL	2243	2257	0.5328	Nonallergen	Nontoxin	99.77%
	LRGTAVMSL	LRGTAVMSLKEGQIN	7052	7066	0.8822	Nonallergen	Nontoxin	99.82%
	LVQMAPISA	LVQMAPISAMVRMYI	2371	2385	1.0016	Nonallergen	Nontoxin	99.86%
	LVQSTQWSL	LVQSTQWSLFFFLYE	3594	3608	1.095	Nonallergen	Nontoxin	99.22%
	PLIVTALRA	PLIVTALRANSAVKL	4125	4139	0.9545	Nonallergen	Nontoxin	99.70%
	VLGLAAIMQ	VLGLAAIMQLFFSYF	2340	2354	0.6624	Nonallergen	Nontoxin	99.71%
	SACVLAAEC	SACVLAAECTIFKDA	2911	2925	0.5526	Nonallergen	Nontoxin	93.56%
	YVLGLAAIM	YVLGLAAIMQLFFSY	2339	2353	0.6002	Nonallergen	Nontoxin	99.87%

^a^the overlapped epitopes between MHCI and MHCII molecules were used once in the vaccine construct as MHCI or MHCII epitopes

### The proposed vaccine construct

**Fig. 2:**
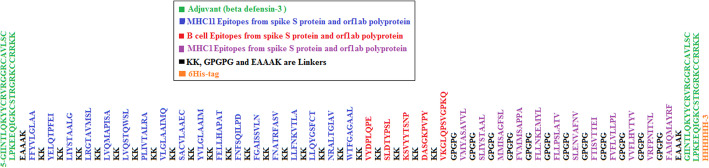
Multi-epitope vaccine design. T helper epitopes (blue colour) and B cell epitopes (red colour) from both spike S protein and orf1ab polyprotein were linked by the short peptide linker KK, while T cytotoxic epitopes (purple color) were linked by GPGPG linker. Human β-defensin-3 (green color) was used as an adjuvant at N and C-terminals and linked by the short peptide EAAAK linkers. C-terminal 6-his was added as his-tag

The total number of proposed epitopes used to built the vaccine construct were five linear B-cell epitopes, 12 T cytotoxic and 18 T helper lymphocytes epitopes from both spike S protin and orf1ab polyprotein. In addition, adjuvants, linkers and His-tag were added to the vaccine construct. Taken together the vaccine construct comprises 526 amino acids (Fig. 2). The vaccine construct was shown to be antigenic in Vaxigen server with score of 0.6194 and nonallergen in the Allertop server.

### Physical and chemical properties of the vaccine construct

The Protparam server demonstrated that the molecular weight of the vaccine construct was 56.37327 k dalton with theoretical isoelectric point value (pI) of 10.19. The total number of negatively (Asp+Glu) and positively (Arg + Lys) charged residues was 18 and 84 respectively. The vaccine construct comprises the 12 amino acids entered in the protein biosynthesis or protein structure. The Extinction coefficients (M^− 1^ cm^− 1^) at 280 nm measured in water was 40,185 assuming all pairs of Cys residues form cystines. The estimated half-life was 30 h (mammalian reticulocytes, in vitro), > 20 h (yeast, in vivo) and > 10 h (*Escherichia coli*, in vivo). The instability index (II) was computed to be 31.25. This classifies the protein as stable. Aliphatic index was 84.39 and the grand average of hydropathicity (GRAVY) was − 0.049 that classified the vaccine construct as hydrophilic.

### BLAST homology assessment

Homology between the sequence of the vaccine and the host proteome sequence demonstrated that the query coverage of the vaccine protein showed only 17% homology to human proteins. This result showed that the predicted vaccine would not implicate in autoimmunity diseases to the host.

### Cluster analysis of the MHC1 restricted alleles

**Fig. 3:**
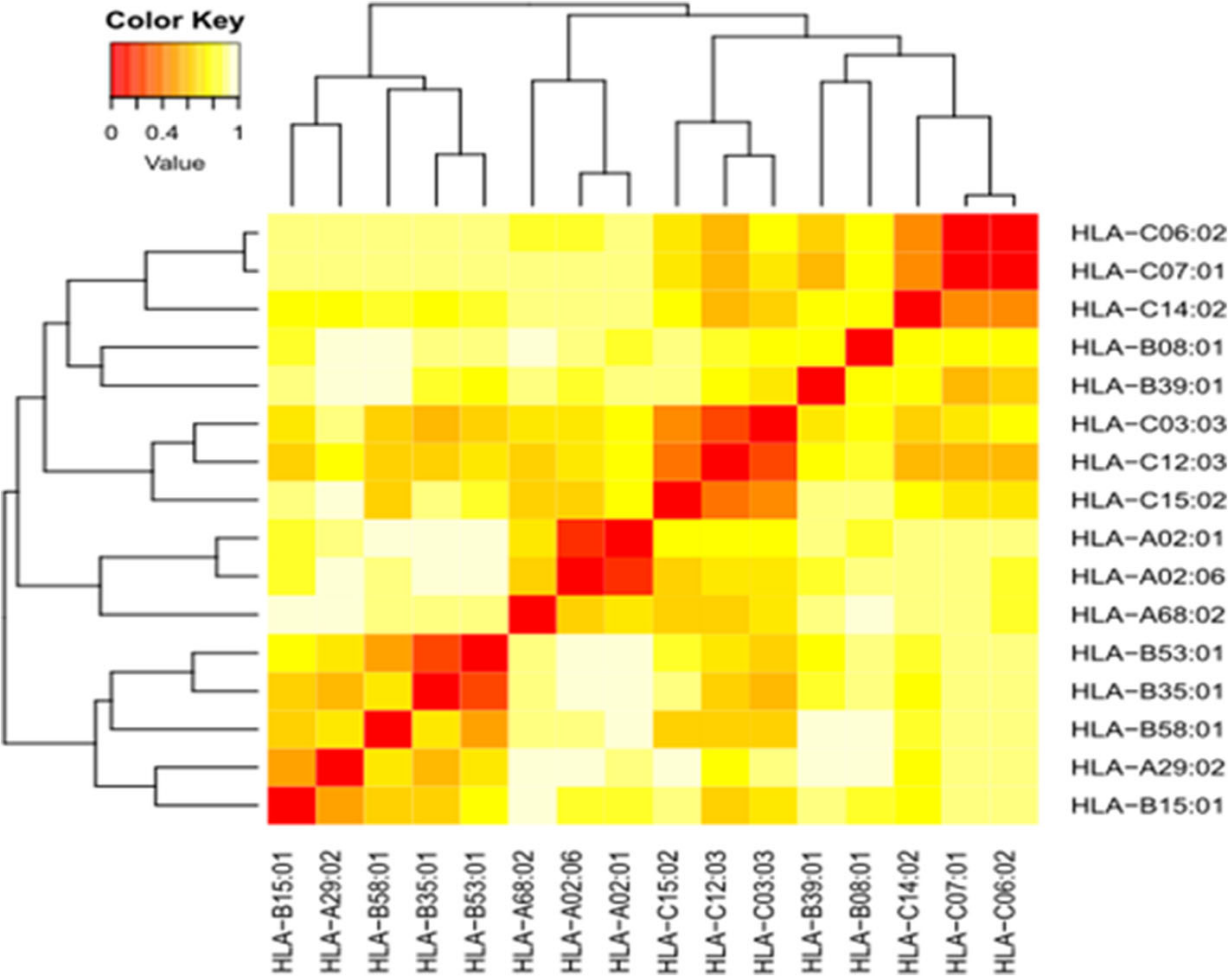
Cluster analysis of the HLA alleles in heat map representation. The red areas indicated strong interaction between HLA alleles while the yellow areas indicated weak interaction

The MHC1 alleles (HLA-A, HLA-B and HLA-C) that interacted with the epitopes from spike S protein and orf1ab polyprotein were clustered by MHCcluster v2.0 server. Sixteen alleles of class I HLA molecules were included in this analysis. Figure 3 showed the cluster analysis of the MHC1 alleles. The figure demonstrated (heatmap) red regions providing strong interaction between the clustering HLA alleles while the yellow regions showed weak allelic interaction between HLA alleles.

### Secondary structure of the vaccine construct

**Fig. 4:**
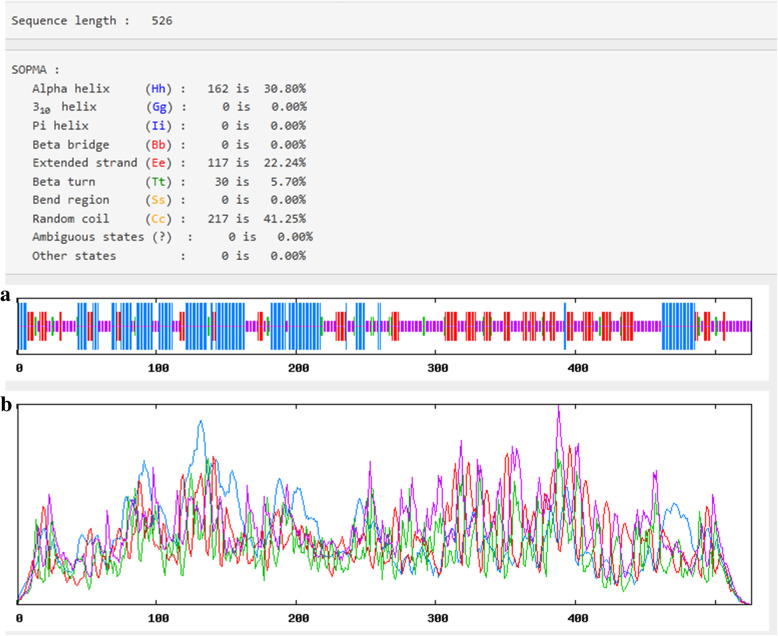
Secondary structure prediction plot of the vaccine construct. Alpha Helices were shown in blue color, while extended strands and beta turns were shown by red and green colours, respectively. The visualization of the prediction (**a**) and the score curves for each predicted state (**b**) were shown

For the secondary structure prediction and as shown in Fig. 4 the vaccine construct demonstrated 30.8% alpha helix, 5.7% beta turn, 22.24% extended strands and 41.25% random coiled.

### Tertiary structure prediction, refinement and adaptation of the vaccine construct

**Fig. 5:**
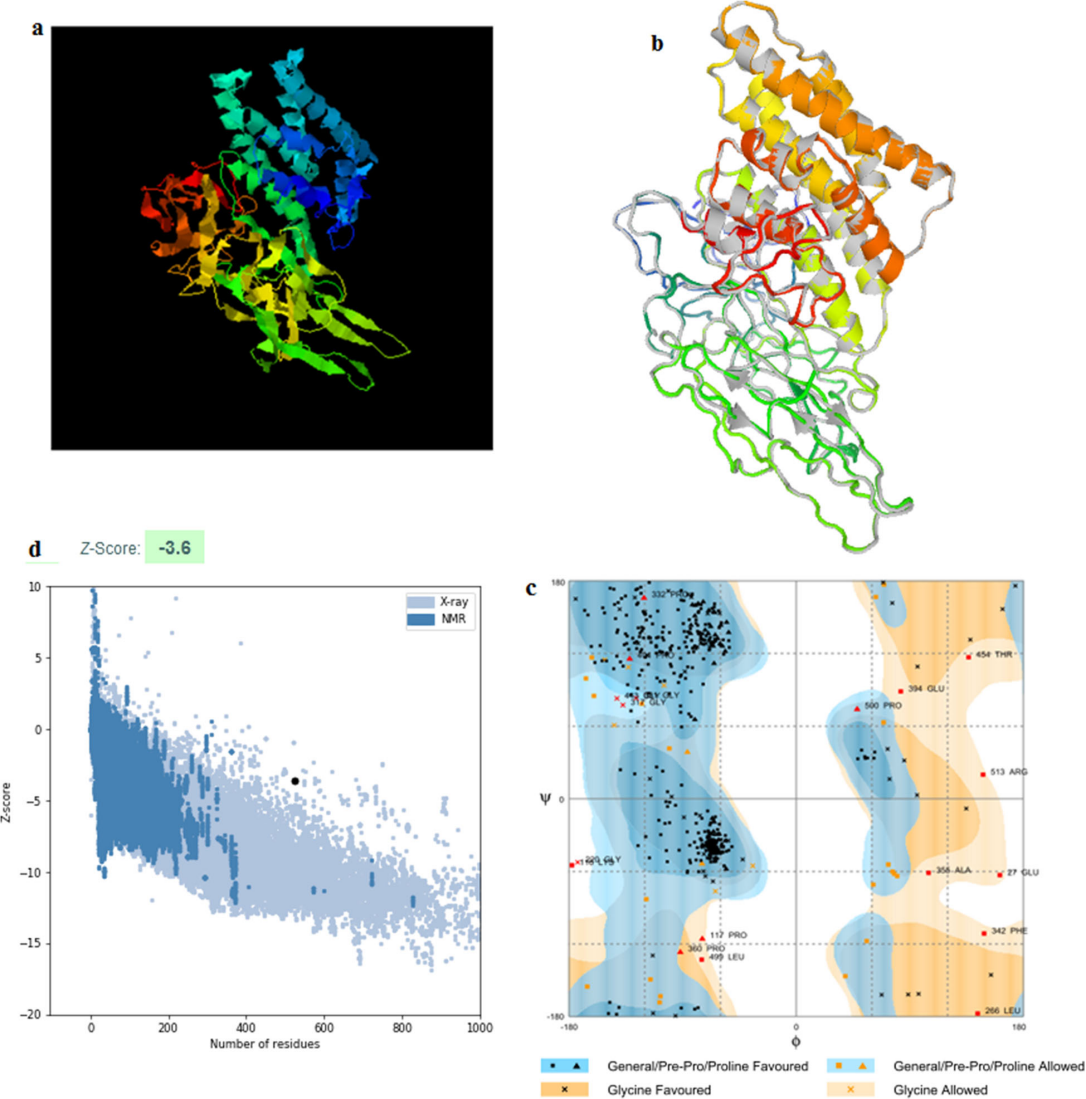
**a** The 3D model of the vaccine construct obtained after homology modelling on I-TASSER. **b** The 3D model was refined in modrefiner and galaxyrefiner and **c** the validated refined model was assessed by Ramachandran plot analysis that demonstrated 91.2%, 5.3% and 3.4% of protein residues in favoured, allowed, and disallowed (outlier) regions respectively. **d** ProSA-server, giving a Z-score of − 3.6

The 3D structure (PDB file) of the vaccine construct that predicted by I-TASSER sever was submitted to ModRefiner and Galaxyrefiner servers to meliorate the quality of predicted 3D modeled structure (Fig. 5). The PDB file was then evaluated by the Ramachandran plot on Rampage. As shown in Fig. 5 the 3D structure of the vaccine construct predicted by I-TASSER server was further analyzed in Ramachandran plot assessment after refinement. Ramachandran plot showed that the number of residues in favoured region was 91.2% and the number of residues in allowed region was 5.3% with only 3.4% of the residues in the outlier region. Moreover proSA server provided Z-score of − 3.6 representing the good quality of the model.

### Solubility and stability (disulfide bonds prediction) of the vaccine construct

**Fig. 6:**
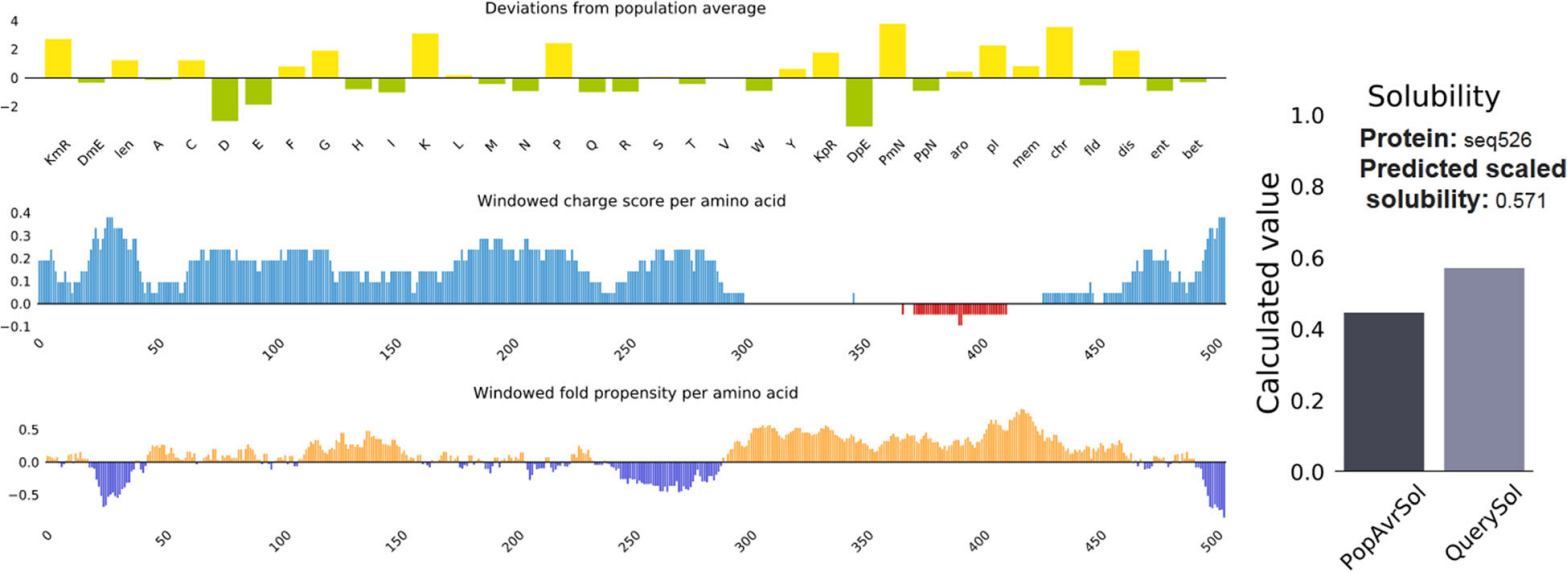
Solubility of the vaccine construct as obtained by protein sol server. The solubility of the vaccine construct was shown to be 0.571 compared to 0.45 of the population average solubility of *E. coli*

**Fig. 7:**
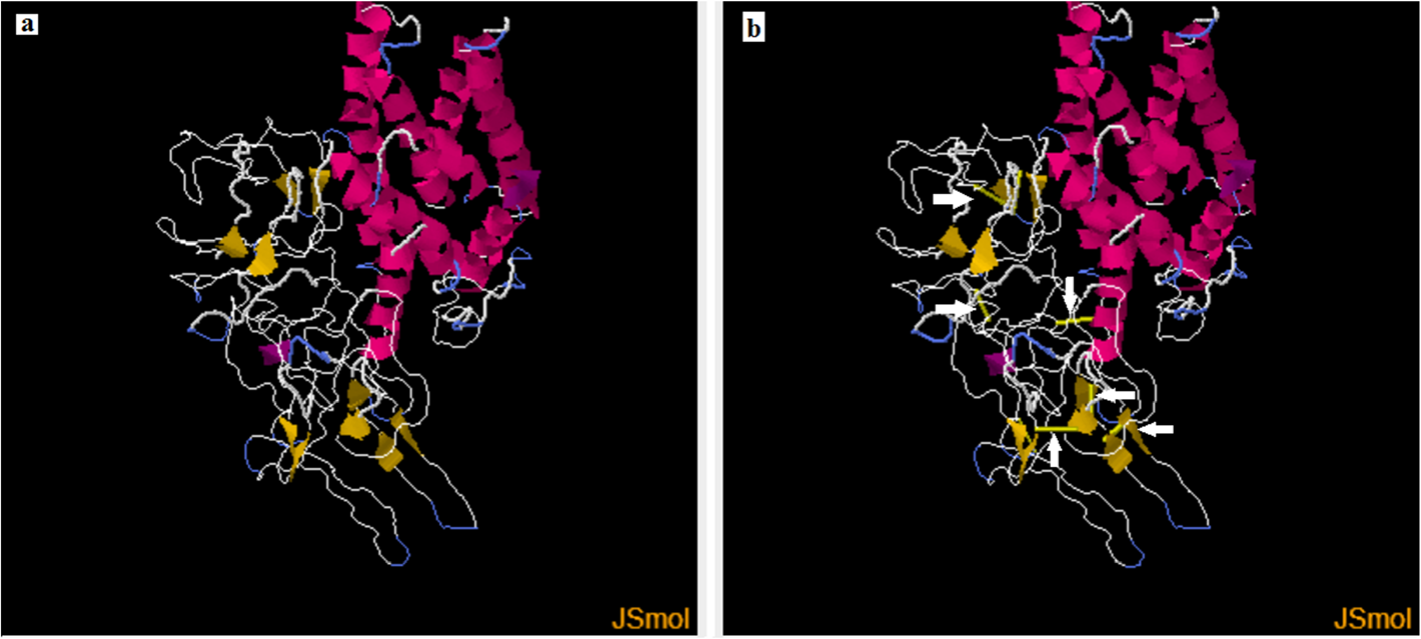
Stability of the vaccine construct by disulfide bond engineering in **a** the original form and **b** the mutant form. Six disulfide bond regions were shown in golden sticky forms indicated by white arrows in the mutant form

Protein-sol server was used to predict the solubility of the vaccine construct. Figure 6 demonstrated that the solubility of the vaccine construct in terms of QuerySol (scaled solubility value) was 0.571. The experimental dataset (PopAvrSol) had a population average of 0.45. Accordingly the solubility of the vaccine construct was larger than 0.45. This result indicated that the vaccine construct is soluble compared to the average solubility of *E. coli* proteins. The solubility of the vaccine construct was further confirmed by SOLpro server. The vaccine construct showed solubility score of 0.873254 greater than the probability of ≥0.5 of the server. For the stability of the vaccine construct, as shown in Fig. 7, residues in the highly mobile region of the protein sequence were mutated with cysteine to perform disulfide engineering. A total 61 pairs of amino acid residues were shown to be probable forming disulfide bonds. Among them only six regions were evaluated to form disulfide bond based on the chi3 residue screening (between − 87 and + 97), B-factor value (ranged 6.950–17.410) and energy value less than 2.5. These six residues were replaced by cysteine residues. The six residue pairs were LYS204-LEU253; SER297-GLY341; VAL315-ALA329; PRO376-PRO451; PRO427-GLY431 and GLY491-LYS519.

### Molecular docking of the vaccine construct with TLR4

**Fig. 8:**
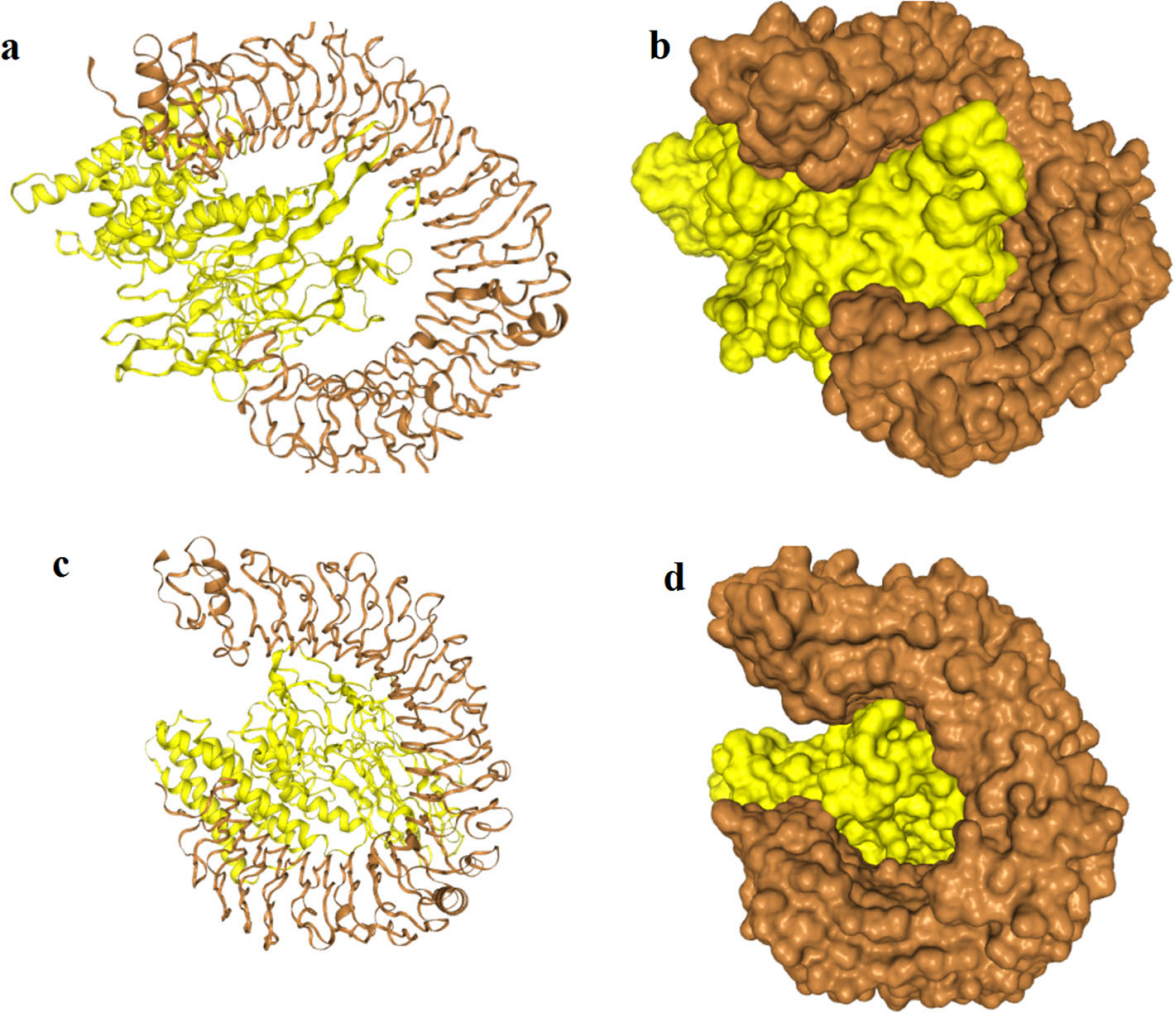
molecular docking between the vaccine construct and the TLR4 chains. The yellow colour represents the vaccine construct while the brown color represents the TLR4 chains. **a** A cartoon structure of the vaccine construct docked with chain A of TLR4 while **b** represents the ball structure. **c** A cartoon structure of the vaccine construct docked with chain B of TLR4 while **d** represents the ball structure

For the docking analysis, the vaccine construct was docked against TLR4 (PDB1D: 4G8A) alpha and beta chains using the HDOCK server. Figure 8 showed that the vaccine construct bound to the TLR4: chain A with attractive binding energy of − 338.68 kcal/ mol. When the vaccine construct docked with TLR4: chain B the attractive binding energy was − 346.89 kcal/mol. The energy score obtained for both A and B chains were the lowest among all other predicted docked complexes showing highest binding affinity. A low (negative) energy indicated a stable system and thus likely binding interaction.

### IFN-γ inducing epitope prediction

**Fig. 9:**
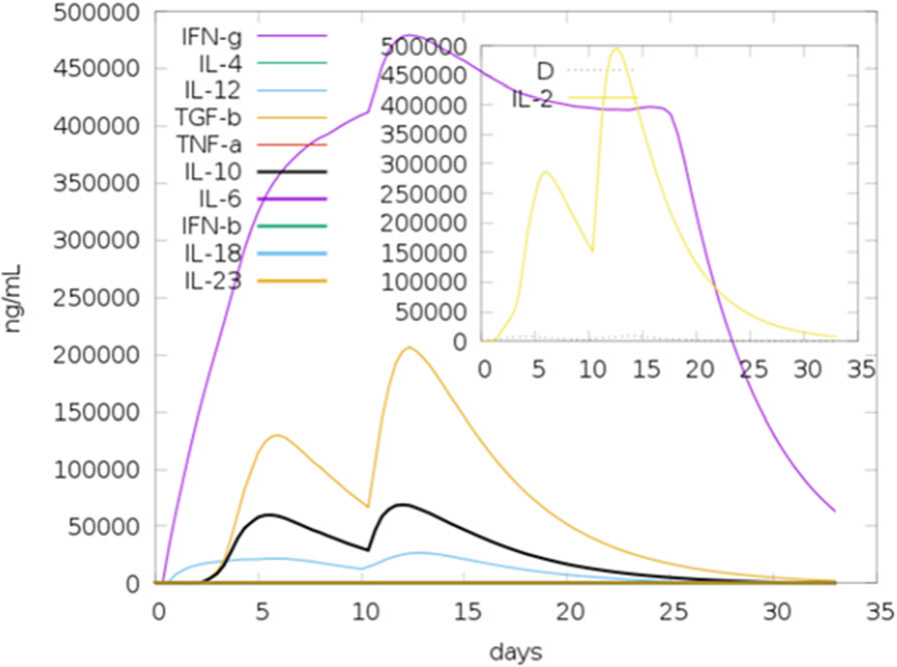
The cytokine levels induced by two injections of the vaccine construct given in interval of 30 days as simulated by C-ImmSim server. The main plot provided the concentration of cytokines and interleukins after the injections. The insert plot showed danger signal together with leukocyte growth factor IL-2 with the Simpson index, D (diversity) shown by the dotted line. The smaller the D value, the lower the diversity

Concerning IFN-γ inducing epitope predictions from the vaccine construct, 412 potential epitopes were predicted from the vaccine construct after removal of the adjuvant. This number includes both +ve and –ve prediction scores. A total of 158 epitopes were predicted to be +ve for inducing IFN-γ with higher score ranging from 1to 7 for 28 epitopes. Figure 9 showed the level of IFN-γ induction during the period of the injections compared to the other cytokines. When the prediction was only performed for the adjuvant, 433 overlapped +ve and –ve epitopes were predicted inducing IFN-γ production. Among them 82 epitopes were predicted positive (+ve). However none of the positive epitopes scored greater than 1. Thus they were considered as IFN-γ non-inducing epitopes.

### Immune simulation of the vaccine construct

**Fig. 10:**
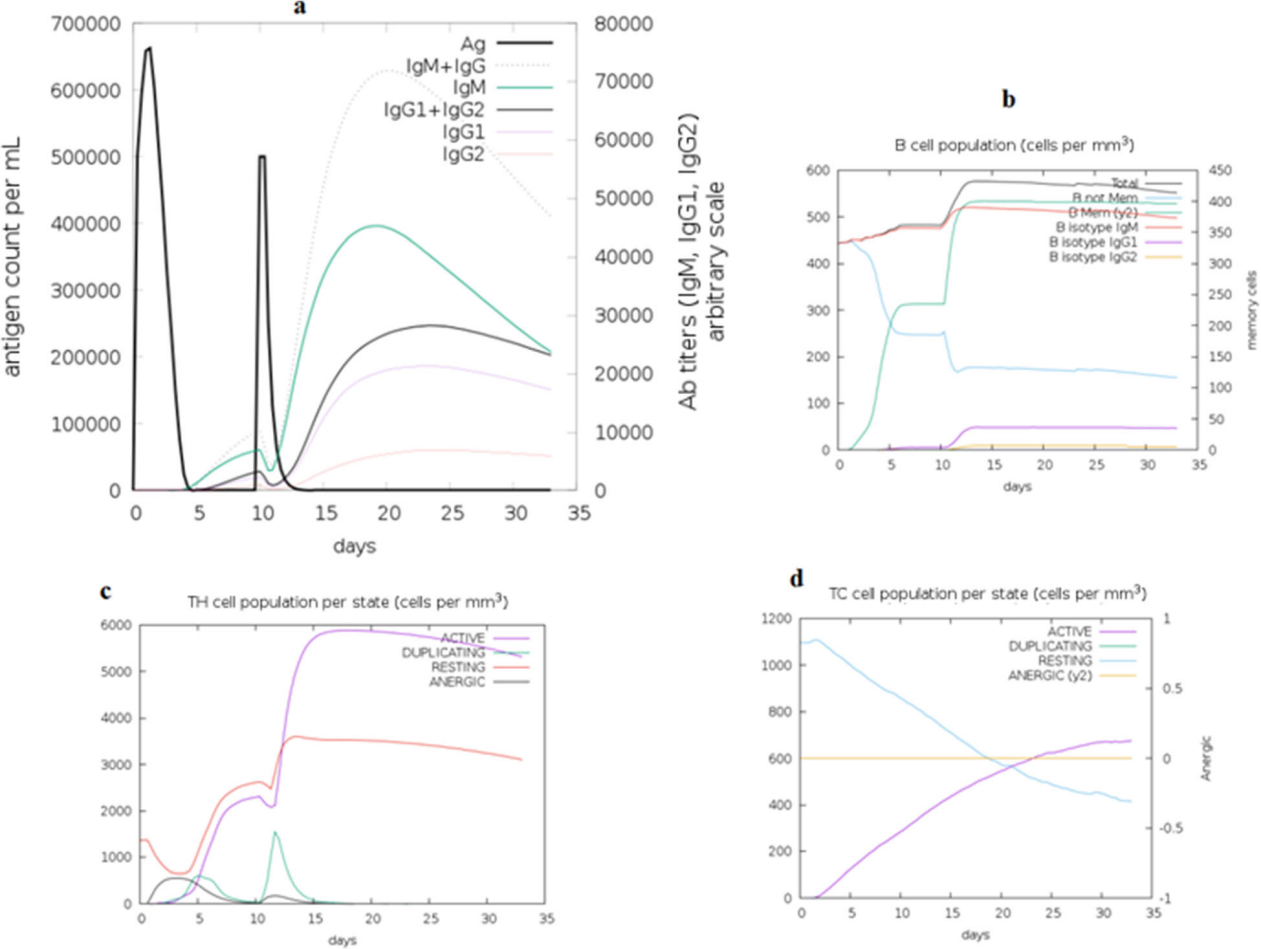
The immune simulation with vaccine construct using C-immsim server. **a** Immunoglobulins production increased in response to exposure to antigen injections with marked decrease in the antigen concentration observed. **b** Showed the B-cell populations with marked increase in the memory and non-memory immunoglobulins. Figure **c** and **d** Showed increased level in the populations of the active T helper and T cytotoxic cells per state after the injections, respectively. The resting state provided cells not exposed to antigen while the anergic state provided tolerance of the T-cells to the antigen exposures

C-ImmSim server was used to mimic the actual immune responses in the body upon exposure to the vaccine construct. Generally the primary immune response occurs as a result of first contact with an antigen and the first antibody produced is mainly IgM, although small amount of IgG are also produced. The amount of antibodies produced depends on nature of antigen and usually produced in low amount. As shown in Fig. 10 the amount of the IgM was markedly started to increase during the first injection of the vaccine construct (antigen) as a primary immune response. Secondary immune response occurs as a result of the second and subsequent exposure to the same antigen and characterized by increased level of IgM and IgG. Also there was marked increased in the level of IgM + IgG and decreased level of the antigen. Moreover there were marked increase in the level of IgM, IgG1 + IgG2, and IgG1 (Fig. 10). This indicated that the antibodies had greater affinity to the vaccine construct (antigen) and would develop immune memory. Consequently, this resulted in increased clearance of the antigen upon subsequent exposures. Concerning the cytotoxic and helper T lymphocytes, high response in the cells populations with corresponding memory development was observed. Most importantly the population of the Helper T lymphocytes remained higher during all exposure time. In the IFN- γ induced epitopes prediction, the results showed that 158 predicted epitopes inducing IFN- γ production without adjuvant. This interpreted the high IFN- γ concentration score compared to the other cytokines. The Simpson index D demonstrated the level of danger when the cytokines level increased that may result in complications during the immune response.

### Codon adaptation and in silico cloning

**Fig. 11:**
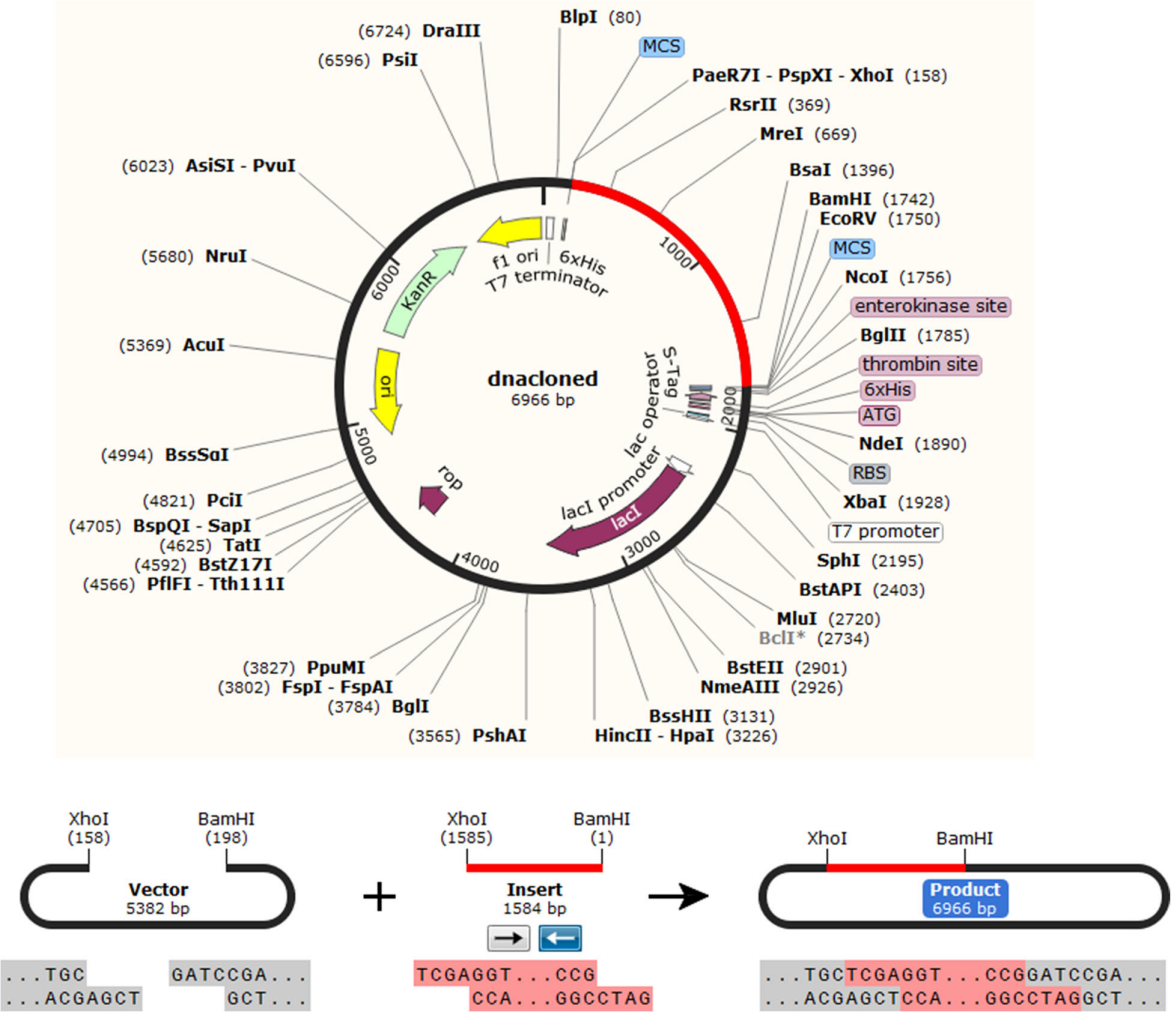
In silico cloning of the final vaccine construct sequence into the pET30a (+) expression vector. The vector was shown in black color, while the red color provided the gene coding for the vaccine construct protein. The DNA sequence of the vaccine construct was typically cloned in the MCS of the vector between BamH1 and Xho1 cutting sites

The protein sequence of the vaccine construct was reversed translated into DNA sequence. Codon adaptation index values (CAI-Value) of the improved DNA sequence was 0.9199, indicating the higher proportion of most abundant codons. The GC-content of the improved sequence was 51.58%, indicating favourable GC content. Figure 11, showed that DNA sequence was cloned into pET28a (+) vector typically at the multiple cloning site (MCS) of the vector after linking BamHI and Xho1restriction enzymes cutting sites sequences to the vicinities of the DNA sequence.

## Discussion

The availability of a safe and effective vaccine for SARS-CoV-2 is well-recognized as an additional tool to contribute to the control of the pandemic. Furthermore enormous challenges and efforts are needed to rapidly develop, evaluate and produce effective vaccine at large scales. In this regard, the Sinovac Biotech has created a new COVID-19 vaccine by growing the novel coronavirus in the VERO monkey cell line and inactivating it with chemicals [31]. The vaccine has protected the rhesus macaques from infection by the new coronavirus. However the vaccine was an old-fashioned formulation consisting of a chemically inactivated version of the virus. Despite that the vaccine produced no obvious side effects in the monkeys and human trials are under processing, but the number of animals tested was too small to yield statistically significant results. Moreover the vaccine may have caused changes that make it less reflective of the ones that infect humans. Another concern is that monkeys do not develop the most severe symptoms that SARS-CoV-2 causes in humans [31]. Generally such kinds of vaccines may have multiple caveats such as the risk of reversion to a more virulent strain of the virus being vaccinated against. Also they may cause severe complications in immunocompromized individuals. In addition to that they are expensive, time consuming and may include unnecessary proteins particles of the virus that provoke immunity, resulting in allergenic and other deleterious immunological responses [32, 33]. Accordingly, recently the focus has shifted towards the development of subunit vaccines as they are associated with better safety profiles and are logistically more feasible [34]. Beside the Sinovac Biotech vaccine, more than 42 vaccines candidates against the pandemic in the clinical trials phases, and some are currently in phase III trials such as Pfizer-BioNTech COVID-19 Vaccine (BNT162b2), ModernaTX, Inc. (mRNA-1273), Sinopharm, CanSino, AstraZeneca and Novavax vaccines [35].

The restrictions on the use of live or attenuated virus vaccines create the need for a safer and effective vaccine. Epitope-based vaccines demonstrated a novel approach for production of a specific immune response and flee the responses against undesirable epitopes in the antigen [36]. Hence, the spike S protein and orf1ab polyprotein were targeted to generate a vaccine construct against SARS-CoV-2 using reverse vaccinology especially enough data about the genomics and proteomics of SARS-CoV-2 become available.

In the current study, the entire viral proteome of SARS-CoV-2 was retrieved from NCBI database. Each protein in the virus was subjected to protein analysis using protparam analysis tool. Moreover the viral proteins were subjected to Vaxijen server to investigate the antigenicity of each protein. All the viral proteins demonstrated antigenicity (scored more than 0.4). Furthermore the viral proteins were examined for the transmembrane helices (TMHs), where the nonstructural orf1ab polyprotein owned the highest number of TMHs. Also the orf1ab polyprotein is the largest protein with 7096 amino acids [2, 22] and plays vital roles in the viral replication, virulence, virus–cell interactions and/or alterations of virus–host response [23]. In the preclinical studies of vaccines against SARS-CoV and MERS-CoV, the spike S protein is the major antigenic determinants that induce neutralizing antibodies [12, 13, 37, 38] and contains the receptor binding domain (RBD) [15–17]. Moreover the majority of the vaccine candidates against SARS CoV were based on the spike S protein and RBD region [12, 13, 15, 20, 21]. Thus these two proteins were targeted for the generation of the vaccine candidates.

In this study a 100% conserved epitopes amongst the screened sequences of spike S protein and orf1ab polyprotein (including those of the new variant strain of Britain, SARS-CoV-2 VUI 202012/01) that could be recognized by B and T lymphocytes to work as vaccine candidates were proposed. For B cell epitopes prediction, the predicted epitopes were obtained using various tools in the IEDB. The predicted B cell epitopes were tested to be linear, surface accessible, antigenic, flexible and hydrophilic using IEDB prediction tools. Furthermore the resulting epitopes were subjected to antigenicity, allergenicity and toxicity analysis. However, only one epitope from the spike S protein and four epitopes from orf1ab polyprotein successfully passed these criteria (Table 1). Thus were proposed as vaccine candidates against B cells. The scarcity of the number of the predicted B cell epitopes may indicate the nonfavourable interaction between the B cells and the virus. Moreover the humoral response from memory B cells can easily be overcome over time by number of antigens, however, cell mediated immunity often elicits long lasting immunity [39, 40].

For T cells, large numbers of epitopes were shown to interact with MHCI and MHCII alleles from spike S protein and orf1ab polyprotein. Epitopes that shown to be antigenic, nonallergic, nontoxic and with high population coverage were elected as a vaccine candidates (Tables 2 and 3). The epitopes _898_FAMQMAYRF_906_ and _800_FNFSQILPD_808_ were previously proposed as vaccine candidates from spike S protein of SARS CoV [21]. Here in this study, the former epitope was also shown to interact with both MHCI and MHC II alleles, while the later epitope interacted only with MHC II alleles of SARS-CoV-2. In addition to that, the two epitopes were located within S2 region (amino acids from 511 to 1190) of the spike S protein that predicted to interfere with fusion of the viral envelope with the host cell and considered as appropriate target for monoclonal antibody development or as vaccine candidates [15]. This result reflected the importance of these two epitopes in SARS-CoV-2 vaccine construction.

For the vaccine to be considered as a global vaccine, the proposed epitopes that constitute the vaccine should interact with most ethnic polymorphic MHC1 and MHC11alleles with high population coverage scores. In this regard the population coverage of the predicted epitopes interacting with T lymphocytes was investigated. The proposed epitopes demonstrated higher affinity to interact with MHC I and MHC II alleles and bound to different sets of alleles with high population coverage scores (Tables 2 and 3). This result indicated that the proposed epitopes as vaccine candidates could cover large population and effectively interacted with the human common alleles worldwide. This result further strengthens the proposed epitopes to work as vaccine candidates against SARS-CoV-2.

One of the most important features of the vaccine protein is not to provide significant similarity or homology to the host proteins. The high similarity between the vaccine as a protein in nature and the host proteome could guide to autoimmune diseases due to molecular mimicry and the chances of cross reactivity [41–43]. In this study the vaccine construct demonstrated less homology (17%) to the human proteins using BLASTp tool providing the vaccine as an excellent candidate with no autoimmunity. Moreover, MHC superfamilies are considered as an essential player in vaccine construction and development as well as drug development. Thus MHC cluster analysis was also performed to assess the functional relationship between MHC1 clustering variants.

To design a vaccine construct, the elected B and T cells epitopes were fused using appropriate specialized spacer (linkers) sequences in order to generate multi-epitopes peptides [44]. The linkers KK and GPGPG were introduced between the selected B and T cells epitopes to generate a sequence with minimal junctional immunogenicity [45–49]. The EAAAK linker was also added between the adjuvants sequences and the fused epitopes in order to reach a high level of expression and improved bioactivity of the fused epitopes [44, 46]. The adjuvants were previously reported as immunomodulator to ameliorate the activity of multiple vaccines [50, 51]. In this regard the β-defensin adjuvant, experimentally, demonstrated an effective immune-stimulation against different kinds of organisms [52–54]. Thus it was used as an adjuvant in the amino and carboxyl terminals of the vaccine construct in this study. Later the vaccine construct was tested for antigenicity and allergenicity and was shown to be antigenic and nonallergic since vaccines with multiple epitopes are often poorly immunogenic and require coupling to adjuvant [44].

The physical and chemical properties showed that the vaccine construct molecular weight was 56.37 k dalton. The computed instability index (II) classifies the protein as stable. Moreover the aliphatic index showed that the protein contains aliphatic side chains, indicating potential hydrophobicity. Moreover the grand average of hydropathicity (GRAVY) was − 0.049 that classified the vaccine construct as hydrophilic. All these characteristics showed that the vaccine protein is thermally stable and therefore suitable as a vaccine against SARS-CoV-2. Furthermore the secondary and tertiary structures of the vaccine construct were evaluated since they are important in vaccine design [44]. Secondary structure analysis showed that the vaccine construct contains alpha helices, extended strands, beta turns and random coiled structures. The 3D structure of the vaccine construct highly ameliorated by the refined software and demonstrated desirable characteristics on Ramachandran plot predictions. Moreover a major problem in structural biology is the recognition of errors in experimental and theoretical models of protein structures [55]. Thus ProSA program was employed to predict the potential structural and modeling errors in the vaccine. The overall quality score was calculated by ProSA program for a specific input structure. The result was displayed in a plot that showed the scores of all experimentally determined protein chains currently available in the Protein Data Bank (PDB) [55]. In this study the predicted vaccine construct demonstrated a Z-score of − 3.6. This indicated that the quality of the overall model is satisfactory as a vaccine candidate against SARS-CoV-2.

Protein solubility and stability have multiple biologically significant functions. For instance the solubility of the overexpressed recombinant protein in the *E. coli* host is one of the important requirements of many biochemical and functional analysis [46, 49]. In this study the solubility of the vaccine construct was measured using protein sol and SOLpro servers. The vaccine construct provided solubility indexes greater than the average probabilities of the servers indicating the solubility of the vaccine construct. Disulfide engineering is important for protein folding and stability. Also structural disulfide engineering decreases the possible number of conformations for a given protein, resulting in decreased entropy and increased thermostability [56–58]. Thus the stability of the vaccine construct was indexed if six residues in the vaccine structure mutated to cysteine.

To strengthen the interaction between the vaccine construct and TLR4, molecular protein-protein docking was performed to explore the binding affinity of vaccine construct with TLR4 chain A and chain B. TLR4 is the key receptor for infectious and noninfectious stimuli that induced a proinflammatory response. TLR4 also plays important role as amplifier of the inflammatory response [59]. In this study the attractive binding energy between TLR4 chains and the vaccine construct demonstrated high binding affinity that expressed in negative binding energy values. Thus this interaction with the TLR4 professionally eliciting a potential protective immune response. Furthermore immune simulation was performed to mimic the typical immune responses. Generally there was marked increase in the immunoglobulins coincided with frequent injection of the vaccine construct. This result indicated the development of memory B cells. Also the level of the active T cytotoxic and T helper lymphocytes were significantly increased supporting the enhancement of humoral and adaptive immune responses. The level of the IFN-γ was also observed high at peak level during the injection times.

Most importantly the expression of the vaccine construct in a suitable *E. coli* expression vector is essential for the production of recombinant proteins [60, 61]. The designed vaccine construct was reverse transcribed and adapted for *E. coli* strain K12 before cloning into pET28a (+) vector. The codon adaptability index (0.9199) and the GC content (51.58%) provided high-level expression of the protein in bacteria. The vaccine construct gene was typically cloned in the vector in the multiple cloning sites. This result provided the successful cloning of the vaccine protein.

## Conclusion

The elimination of the pandemic is coincided with development of novel control measures to combat the infection of SARS-CoV-2. In this study a unique vaccine construct (multiepitopes) was generated from spike S protein and orf1ab polyprotein against B and T lymphocytes via various bioinformatics tools. This proposed vaccine construct could potentially provide protection against the pandemic SARS-CoV-2 and/or used as complementary tool to eliminate the infection. Therefore, the present study might assist in developing a suitable therapeutics protocol to combat SARS-CoV-2 infection.

## Methods

### The retrieval of the viral whole proteome

The entire viral proteome of SARS-CoV-2 (COVID-19) was retrieved from National Center For Biotechnology Information (NCBI) at (https://www.ncbi.nlm.nih.gov/genome/browse/#!/proteins/86693/757732%7CSevere%20acute%20respiratory%20syndrome%20coronavirus%202/). The virus demonstrated 12 proteins. These 12 proteins accession numbers, lengths and names were shown in Table 4.

**Table 4:** Physical and chemical properties, antigenicity and number of the predicted transmembrane helices of SARS CoV-2 proteins

Viral protein	Accession number	Molecular weight (Dalton)	Instability index^b^	Aliphatic index	Theoretical pI	No amino acids	Extinction coefficient	GRAVY^c^	Vaxijen antigenicity^d^	TMHs
orf1ab polyprotein	YP_009724389.1	794,057.79	33.31	86.87	6.32	7096	942,275	−0.07	0.4624	14
orf1a polyprotein	YP_009725295.1	489,988.91	34.92	88.99	6.04	4405	552,175	−0.023	0.4787	14
Surface glycoprotein	YP_009724390.1	141,178.47	33.01	84.67	6.24	1273	148,960	−0.079	0.4646	1
orf3a protein	YP_009724391.1	31,122.94	32.96	103.42	5.55	275	58,705	0.275	0.4945	3
Envelope protein	YP_009724392.1	8365.04	38.68	144	8.57	75	6085	1.128	0.6025	1
Membrane glycoprotein	YP_009724393.1	25,146.62	39.14	120.86	9.51	222	52,160	0.446	0.5102	3
orf6 protein	YP_009724394.1	7272.54	31.16	130.98	4.6	61	8480	0.233	0.6131	0
^a^orf7a protein	YP_009724395.1	13,744.17	48.66	100.74	8.23	121	7825	0.318	0.6441	1
orf7b protein	YP_009725296.1	5180.27	50.96	156.51	4.17	43	7115	1.449	0.6025	1
orf8 protein	YP_009724396.1	13,831.01	45.79	97.36	5.42	121	16,305	0.219	0.6502	0
Nucleocapsid phosphoprotein	YP_009724397.2	45,625.7	55.09	52.53	10.07	419	43,890	−0.971	0.5059	0
^a^orf10 protein	YP_009725255.1	4449.23	16.06	107.63	7.93	38	4470	0.637	0.7185	0

*THMs* Transmembrane helices

^a^the protein contains no tryptophan

^b^instability index < 40 considered the protein stable

^c^GRAVY negative sign indicated the protein is hydrophilic

^d^the threshold for the Vaxijen antigenicity is 0.4

### Physical and chemical properties of the viral proteins, antigenicity and transmembrane topology

ProtParam (http://web.expasy.org/protparam/) is a tool allowed the computation of various physical and chemical parameters for a given protein sequence. Each protein was subjected to Protparam server for the physiochemical properties and the computed parameters covered the molecular weight, theoretical pI, amino acid composition, extinction coefficient, instability index, aliphatic index and grand average of hydropathicity (GRAVY). Moreover the VaxiJen v2.0 server at (http://www.ddg-pharmfac.net/vaxijen/) which based on auto- and cross-covariance transformation of protein sequences into uniform vectors of principal amino acid properties was used to analyze the potent antigenicity of each protein of SARS-CoV-2. The viral proteins were further analyzed for transmembrane topology using TMHMM (http://www.cbs.dtu.dk/services/TMHMM/). Proteins that demonstrated best physiochemical properties, antigenicity and transmembrane topologies were allowed for further analysis. In this essence, as shown in Table 4 only the first three proteins in the table demonstrated best physical and chemical properties despite all the viral proteins were shown to be antigenic by VaxiJen v2.0 passing the threshold of (0.4) and contained varied numbers of TMHs. It is noteworthy that the viral orf1ab polyprotein and orf1a polyprotein upon alignment the later was shown to be partial from the former (orf1ab). Accordingly, the spike S protein and orf1ab polyprotein were targeted for prediction of epitopes as vaccine candidates that could elicit both B and T lymphocytes.

### Protein sequences retrieval of spike S proteins and orf1ab polyprotein

A set of available 714 orf1ab polyproteins at (https://www.ncbi.nlm.nih.gov/protein/?term=orf1ab+polyprotein+%5BSevere+acute+respiratory+syndrome+coronavirus+2%5D) and 9 proteins of spike S glycoproteins at (https://www.ncbi.nlm.nih.gov/protein/?term=spike+S+protein+severe+acute+respiratory+syndrome+2+) of SARS-CoV-2 were retrieved from the NCBI. These sequences were retrieved in FASTA format and further used for epitopes conservancy among the retrieved strains. The spike S protein (id= QQL92050.1) and orf1ab protein (id= QQL92048.1) of the new variant strain SARS-CoV-2 VUI 202012/01(MW450666.1) that was recently identified in Britain was also included in the epitopes conservancy analysis.

### Sequence alignment and determination of the conserved regions

The retrieved protein sequences of spike S protein and orf1ab polyprotein were further aligned to obtain conserved epitopes using multiple sequence alignment (MSA) tools, Clustal W, embedded in the BioEdit program, version 7.0.9.0 [62]. MSA analysis was performed to analyze 100% conserved epitopes amongst the screened sequences of spike S protein and orf1ab polyprotein.

### B-cell epitopes prediction

B-cell epitopes are antigenic determinants recognized by the immune system and represent the specific piece of the antigen to which B lymphocytes bind. These play a vital role in vaccine design. The Immune Epitope Data Base web server (IEDB) at (https://www.iedb.org/) was used for prediction of B cell epitopes from spike S protein and orf1ab polyprotein. A collection of methods to predict B cell epitopes based on sequence characteristics of the antigen using amino acid scales and hidden Markov Models (HMMs) were used. For instance; Linear B-cell epitopes were predicted using BepiPred linear epitopes prediction tool [63–65]. Emini Surface Accessibility prediction method was used to obtain surface epitopes [66]. The antigenicity of the predicted epitopes was performed using Kolaskar and Tongaonkar Antigenicity prediction tools [67]. For prediction of epitopes flexibility and hydrophilicity, the Karplus and Schulz flexibility and Parker hydrophilicity prediction tools were used [68, 69].

### Cytotoxic T lymphocytes epitopes prediction

The epitopes binding analysis to Major Histocompatibility Complex class I molecules (MHC class I) from spike S protein and orf1ab polyprotein was performed using IEDB MHC I tools at (http://tools.iedb.org/mhci/). The MHC I epitope molecules that interacted to T lymphocytes was subjected to multiple steps. This prediction method used an amino acid sequence, or set of sequences and determined each subsequence’s ability to bind to a specific MHC class I molecule. The binding of the fragmented peptides to MHC molecules step was predicted by Artificial Neural Network 4.0 (ANN 4.0) method. Prior to the prediction, all lengths of epitope was set as 9mers and all the conserved epitopes that bound to alleles at score of ≤100 half-maximal inhibitory concentration (IC50) were subjected for further analysis [70–73].

### Helper T-lymphocytes epitopes prediction

Analysis of peptides binding to MHC II molecules from spike S protein and orf1ab polyprotein was assessed by the IEDB MHC II prediction tool at (http://tools.iedb.org/mhcii/result/). For MHC II binding predication, human allele’s reference sets (human HLA-DR, HLA-DQ, HLA-DP) were used. MHC II groove has the ability to bind different lengths peptides that makes prediction more difficult and less accurate. Thus Neural Network align (NN-align 2.3; Net MHCII 2.3) was used to identify both the binding affinity and MHCII binding core epitopes. Prior to the prediction, the length of peptide was set as 15mers (15 amino acids) and all the conserved epitopes that bound to alleles at score of score of ≤1000 half-maximal inhibitory concentration (IC50) were subjected for further analysis [74].

### Antigenicity, allergenicity and toxicity of the predicted epitopes

Analysis of the antigenicity, allergenicity and toxicity of the predicted epitopes from spike S protein and orf1ab polyprotein for B and T lymphocytes, was performed using multiple prediction tools. The predicted epitopes were submitted to the VaxiJen v2.0 server for antigenicity prediction. The threshold of VaxiJen v2.0 server was set to the default threshold (0.4). Epitopes that demonstrated antigenicity were further investigated for their allergenicity using AllerTOP server [75]. Epitopes found to be antigenic and non-allergenic were further assessed for toxicity by ToxinPred server [76].

### Population coverage

Epitopes that interacted with MHC I and MHC II from spike S protein and orf1ab polyprotein were subjected to population coverage analysis after they were shown to be antigenic, nonallergic and nontoxic. The population coverage was investigated against the whole world using IEDB population coverage tool at (http://tools.iedb.org/tools/population/iedb_input).

### Vaccine construction (multiepitopes vaccine)

Epitopes that passed the criteria of B cell epitopes prediction, epitopes with high allelic interaction and best population coverage scores against cytotoxic and helper T lymphocytes were used to generate the vaccine construct. Epitopes that overlapped in both MHC I and MHC II were used once in the vaccine construct as MHC I or MHC II epitopes. The vaccine construct was generated as previously described [45, 46] with minor modifications. The GPGPG linker was used to fuse the B cell and T helper predicted epitopes. While KK linker was used to link the epitopes of T cytotoxic lymphocytes. EAAAK linker was used to link the epitopes with the human β-defensin (uniprot entry Q5U7J2) that was used as an adjuvant on the amino and carboxyl terminals to ameliorate the immunogenicity of the vaccine construct [77]. β-defensin was shown to induce potential immunogenic responses similar to natural immune responses [77]. Linkers were shown to assist in enhancing expression, stability and folding of the protein by separating the functional domains [43, 78].

### Physical and chemical properties of the vaccine construct

The vaccine construct from predicted epitopes was analyzed for the physical and chemical properties using Protparam analysis tool. The computed parameters covered the molecular weight, theoretical pI, amino acid composition, extinction coefficient, estimated half-life, instability index, aliphatic index and Grand average of hydropathicity (GRAVY).

### BLAST and assessment of vaccine protein against the human proteins

Protein BLAST was performed to find similarity or homology between the vaccine protein construct with the human proteome via NCBI BLASTp [79, 80]. The aim behind this homology analysis was to avoid autoimmunity that might be caused due the homology between the vaccine and human proteins**.** The search in Protein BLAST was limited to records that include: Homo sapeins (taxid: 9606). The result of the homology score must be no or least homology (< 40%) to the human proteome [41].

### Cluster analysis of the MHC1 restricted alleles

The MHC genomic region in most species is extremely polymorphic. The human MHC genomic region (HLA) is extremely polymorphic comprising several thousand alleles, many encoding a distinct molecule. The potentially unique specificities remain experimentally uncharacterized for the vast majority of HLA molecules [81]. The MHCcluster server is a tool to functionally cluster MHC class I molecules (MHC I) based on their predicted binding specificity was used [81]. The functional relationship between the allelic variants is presented as a phylogenetic tree and/or heat-map between MHC variants [45, 81].

### Secondary structure prediction

Self-optimized prediction method (SOPMA) at (https://npsa-prabi.ibcp.fr/cgi-bin/npsa_automat.pl?page=/NPSA/npsa_sopma.html) was used to predict alpha helix, coiled structures and beta sheets in the secondary structure of the vaccine construct [82].

### Tertiary structure prediction, refinement and validation

The vaccine construct sequence was submitted to I-TASSER protein folding recognition server [83]. The server is an active development with the goal to provide the most accurate protein structural and functional predictions using state-of-the-art algorithms. The PDB file obtained by I-TASSER was submitted to ModRefiner [84] and GalaxyWEB [85, 86] web servers for protein structure prediction, refinement, and related methods. The refinement was performed to ameliorate the physical quality of the structure. The refined protein structure was further validated through Ramachandran plot assessment at RAMPAGE [87, 88]. Moreover the refined PDB file obtained by I-TASSER server was analyzed by ProSA server for structure potential errors [55]. ProSA-web Z-score is depicted in a plot, which includes the Z-score of experimentally determined structures deposited in PDB.

### Solubility and stability (disulfide bonds prediction) of the vaccine construct

Protein-sol (https://protein-sol.manchester.ac.uk/) is a web based suite of theoretical calculations and predictive algorithms for understanding protein solubility [89]. The solubility of the vaccine construct was analyzed compared to solubility in databases. The server predicted the solubility of proteins in terms of QuerySol (scaled solubility value). The experimental dataset (PopAvrSol) had a population average of 0.45. Accordingly the protein solubility scores larger than 0.45 is expected to be soluble compared to the average solubility of *E. coli* proteins from the experimental solubility dataset and vice versa [45, 90].

The solubility of the vaccine construct was further analyzed by SOLpro server (http://scratch.proteomics.ics.uci.edu/) to predict the solubility upon overexpression.

SOLpro predicts solubility based on the probability of ≥0.5. Thus soluble protein scores ≥0.5 and insoluble protein scores < 0.5. For stability, the disulfide bonds strengthen the geometric conformation of the vaccine construct and provided significant stability. The Disulfide by Design 2.0 (DbD2) is a web-based tool for disulfide engineering in proteins was used to design disulfide bonds in the vaccine construct [91]. For a given protein structural model to predict disulfide bonds, all residue pairs are rapidly assessed for proximity and geometry consistent with disulfide formation, assuming the residues were mutated to cysteine.

### Molecular docking of the vaccine construct with TLR4 (protein-protein docking)

Protein–protein and protein–DNA/RNA interactions play a fundamental role in a variety of biological processes. Determining the complex structures of these interactions is valuable, in which molecular docking has played an important role. HDOCK server that used protein-protein and protein-DNA/RNA docking based on a hybrid algorithm of template-based modeling and ab initio free docking was used to dock the vaccine construct with human Toll-Like Receptor4 (TLR4) [92]. The vaccine construct PDB file was submitted to the server with TLR4 (PDB ID: 4G8A) as a receptor for the docking process.

### IFN-γ inducing epitope prediction

IFNepitope server (http://crdd.osdd.net/raghava/ifnepitope/scan.php) is a module designed for predicting Interferon gamma (IFN-γ) inducing regions in a protein or antigen by generated all possible overlapping peptides (of length or window selected by user) from the protein or antigen. The server identifies best antigenic regions or IFN epitope in a query antigen sequence that can induce IFN-γ. Interferon gamma (IFN-γ), has an impact on the adaptive and innate immune responses, provokes immune system cells and raised response to MHC antigens. The prediction process was performed as previously described [46, 93] with minor modification. The length of the designed peptide was set to15-mers IFN-γ epitopes. The prediction was performed by Supportive Vector Machine approach.

### Immune simulation

To further characterize the immunogenicity and immune response profile of the vaccine construct, an in silico immune simulations were conducted using the C-ImmSim server (http://150.146.2.1/C-IMMSIM/index.php) [94]. Two injections with vaccine construct were given at intervals of 30 days. The Simpson index, D (a measure of diversity) was interpreted from the plot.

### Codon adaptation and in silico cloning

Codon adaptation and in silico cloning were performed in order to express the final vaccine construct in the *E. coli* (strain K12) host since codon usage optimization demonstrated differences between human and *E. coli* strain. The purpose of this cloning was to guarantee the expression of the vaccine construct in the selected host. Java Codon Adaptation Tool (JCAT) server (http://www.prodoric.de/JCat) was firstly used for the reverse translation of the protein sequence of the vaccine construct into DNA sequence. The rho independent transcription termination, prokaryote ribosome binding site and cleavage site of restriction enzyme were avoided [46]. In the JACT, codon adaptation index (CAI) score is 1.0 but > 0.8 is considered a good score [95]. The favourable GC content of a sequence ranged between 30 and 70%. Secondly, BamHI and Xho1restriction enzymes cutting sites sequences were introduced to the DNA sequence obtained by (JCat) server at the N-terminal and C-terminal vicinities, respectively. The SnapGene restriction cloning module [46, 47] was used to insert the DNA sequence into pET28a (+) vector between the BamHI and Xho1.

## Data Availability

The datasets during and/or analyzed during the current study available from the corresponding author on reasonable request.

## Abbreviations

SARS-CoV-2: Severe acute respiratory syndrome coronavirus-2
SARS-CoV: Severe acute respiratory syndrome coronavirus
COVID-19: Coronavirus disease 2019
RBD: Receptor binding domain
ACE2: Angiotensin converting enzyme 2
PLpro: Papain-like protease
3CLpro: 3C-like protease
NSP: Nonstructural proteins
IEDB: Immune Epitope Data Base web server
MHC 1: Major Histocompatibility Complex class I
MHC II: Major Histocompatibility Complex class II
HLA: Human leucocyte antigen
ANN: Artificial Neural Network
NN-align: Neural Network align
IC50: Half-maximal inhibitory concentration
pI: Isoelectric point
GRAVY: Grand average of hydropathicity
TLR4: Toll like Receptor 4
BLASTp: Basic local alignment search tool for protein
3D structure: Three dimensional structure
PDBfile: Protein Data Bank file
QuerySol: Query solubility
PopAvrSol: Population average solubility
CAI: Codon adaptation index
JCAT: Java Codon Adaptation Tool
MCS: Multiple cloning site
TMHs: Transmembrane helices
TMHMM: Transmembrane helices prediction method based on a hidden Markov Model
NCBI: National Center for Biotechnology Information
MSA: Multiple sequence alignment
SOPMA: Self-optimized prediction method
